# Perceived Burdensomeness and the Wish for Hastened Death in Persons With Severe and Persistent Mental Illness

**DOI:** 10.3389/fpsyt.2020.532817

**Published:** 2021-01-12

**Authors:** Julia Stoll, Christopher James Ryan, Manuel Trachsel

**Affiliations:** ^1^Institute of Biomedical Ethics and History of Medicine, University of Zurich, Zurich, Switzerland; ^2^Discipline of Psychiatry, Westmead Clinical School and Sydney Health Ethics, University of Sydney, Sydney, NSW, Australia; ^3^Clinical Ethics Unit, University Hospital Basel and University Psychiatric Clinics Basel, Basel, Switzerland

**Keywords:** suicide, euthanasia, depression, schizophrenia, anorexia nervosa, ethics, autonomy, mental competency

## Abstract

**Background:** In several European countries, medical assistance in dying (MAID) is no longer confined to persons with a terminal prognosis but is also available to those suffering from persistent and unbearable mental illness. To date, scholarly discourse on MAID in this population has been dominated by issues such as decision-making capacity, uncertainty as to when a disease is incurable, stigmatization, isolation, and loneliness. However, the issue of perceived burdensomeness has received little attention.

**Objective:** The study explores the possible impact of perceived burdensomeness on requests for MAID among persons with severe and persistent mental illness (SPMI).

**Method:** Using the method of ethical argumentation, we discuss the issue of access to MAID for persons with SPMI and perceived burdensomeness.

**Conclusion:** Perceived burdensomeness may be a contributing factor in the wish for hastened death among persons with SPMI. MAID is ethically unsupportable if SPMI causes the individual to make an unrealistic assessment of burdensomeness, indicating a lack of decision-making capacity in the context of that request. However, the possibility that some individuals with SPMI may perceive burdensomeness does not mean that they should be routinely excluded from MAID. For SPMI patients with intact decision-making capacity who feel their life is not worth living, perceived burdensomeness as a component of this intolerable suffering is not a sufficient reason to deny access to MAID.

## Introduction

Despite all best efforts to treat mental illness and promote recovery, some patients develop a *severe and persistent mental illness* (SPMI) that overwhelms their life, such as severe treatment-refractory major depressive disorder, extremely disabling chronic schizophrenia, or anorexia nervosa [(1, 2); for a systematic review of definitions of SPMI, see (3)]. Patients with SPMI should be offered all treatment options, including palliative care (4). In addition, however, it is important to interrogate the ethics of allowing individuals with SPMI to access *medical assistance in dying* (MAID). This encompasses both euthanasia, where the physician administers the lethal drug, and physician-assisted dying, where the physician only prescribes the drug, and the patient actively takes it [e.g., (5)]. Although reforms to facilitate MAID are motivated mainly by the unbearable suffering of terminally ill patients and are confined to that population, similar ethical arguments can be applied to those with no terminal condition. As outlined below, some jurisdictions already allow persons with SPMI to access MAID. However, many do not, and some authors have proposed that MAID should be extended in all jurisdictions to psychiatric conditions (6, 7). According to Schuklenk and van de Vathorst (7), “Jurisdictions that are considering, or that have, decriminalized physician assisted dying are discriminating unfairly against patients suffering from treatment-resistant depression if they exclude such patients from the class of citizens entitled to receive assistance in dying” (p. 577).

To begin, it is useful to briefly summarize the current legal situation regarding MAID in various jurisdictions. In the Netherlands, existing legislation exempts physicians participating in MAID (including euthanasia) from criminal liability in nonterminal cases where the physician believes, among other things, that the patient's suffering is “*lasting and unbearable*” (8–10). In Belgium, mental illness is explicitly recognized as permissible grounds for MAID (including euthanasia) when the suffering is constant and cannot be otherwise alleviated (9–12). In Luxembourg, MAID (including euthanasia) is permitted if an individual has a severe and incurable illness and “*constant unbearable physical or psychological suffering*” if the individual's request for MAID is stable over time (10–13). In Switzerland, MAID (assisted suicide but not euthanasia) is legally unrestricted for patients with serious, incurable, and chronic mental illness [(10, 12, 14)^1^].

In those jurisdictions that allow MAID in cases of SPMI, one important criterion is that the patient must be experiencing intolerable physical or psychological suffering. However, participants often differ about the meaning of “*intolerable suffering*” (15). Most scholars have argued that suffering is purely subjective and can only be appraised by the patient (2, 16, 17), and unbearable suffering has been defined as “a subjective experience of suffering that is so serious and uncontrollable that it overwhelms ones bearing capacity […]” [(18), p. 2]. This is not generally limited to physical causes of suffering such as pain or other somatic symptoms but includes psychological symptoms or social difficulties (2, 16, 18). It has been previously argued that, on this view, MAID may be in the patient's best interest and should therefore be an available option (19). The core argument here is that maximizing the patient's autonomy in choosing his/her own way of dying also minimizes their suffering (20).

*Perceived burdensomeness* has been defined as the “[…] perception that one is a burden or drain on significant others […]” [(21), p. 631] or a burden on society (21, 22). To date, this issue has received little attention in the scholarly discourse on MAID, probably because the concept of perceived burdensomeness is difficult to grasp, as it involves both the patient's subjective feelings and the objective external reality, which may conflict. As the issue is also emotionally charged, it is all the more important to discuss the associated ethical considerations as objectively as possible and without prejudice.

Perceived burdensomeness is central to MAID decision making from two perspectives. First, the patient might choose MAID because they perceive themselves to be a burden to relatives or society and wish to free others from that burden. As there may be some felt pressure to do so, choosing MAID might not be a completely autonomous decision. Second, the patient's perception that they are a burden for caregivers may form part of the intolerable suffering that is prerequisite of legal eligibility for MAID. What, then, distinguishes perceived burdensomeness from other aspects of intolerable suffering? In the first place, it is important to note that both are subjective and therefore difficult to assess objectively. On the one hand, perceived burdensomeness is a purely psychological rather than a partly physical aspect of intolerable suffering, making it more difficult to grasp. On the other hand, perceived burdensomeness depends on external factors as well as on the individual. This is a significant source of difficulty, as a person's preference for MAID should not be driven by external factors.

Among the internal aspects of perceived burdensomeness, the individual's sense of autonomy is very important, and this may be violated by a constant need for care. Even with the best of care, this aspect of perceived burdensomeness cannot be changed from without, as it is an element of SPMI precisely because of its *persistence*. We assume here that a chronic and persistent disorder of this kind may entail a chronic need for care and dependence, unlike an acute psychiatric situation in which that need is often transient, with the prospect of more rapid improvement and at least partial independence. It seems likely that dependence or need for care will be easier to bear if it is transient rather than permanent or ongoing. At the same time, it should be noted that a patient may perceive themselves as a burden without experiencing this as part of their intolerable suffering. In other words, they may recognize this but can accept it, in which case perceived burdensomeness would not contribute to their wish for MAID.

To address the question of allowing access to MAID for persons with SPMI and perceived burdensomeness, we have adopted the method of ethical argumentation. An argument is ethical when some of its premises, as well as its conclusion, make normative, value-laden claims as opposed to being merely descriptive. This distinction between *normative* and *descriptive* dates back to David Hume, who “[…] noted a categorical difference between the moments people talk about how things are, and the moments people talk about how things ought to be […]” [(23), p. 18].

The rest of the paper is organized as follows: After first outlining the relevance of perceived burdensomeness for some individuals who express a desire for MAID, we go on to examine the widely held view that persons with SPMI should be routinely excluded from MAID because of the possible influence of perceived burdensomeness. The final section includes a brief summary and a discussion of some practical implications.

## Perceived Burdensomeness as an Influence on the Desire for Hastened Death

Concern has previously been expressed that a causal factor in some suicides is that the person in question had come to believe they were a burden to others (21, 22, 24–27). The feeling or fear of being a burden to others is often an important end-of-life concern (10, 28), not only among the terminally ill but also among healthy older adults (29). Perceived burdensomeness may also play an important role in the wish to die or the wish for hastened death (28, 30–37), and in requests for MAID (9, 25, 28, 30, 32, 36–38), extending even to a perceived “duty to die” (36, 39). While most of these studies focused mainly on patients with terminal physical illnesses, Groenwoud et al.'s (38) survey of psychiatrists in the Netherlands found that being a burden to others might also motivate requests for MAID among patients with mental illness.

Although rarely alluded to in the academic literature, the concern that perceived burdensomeness might be a causal factor in suicide sometimes informs arguments by clinicians and non-academics against MAID for patients with SPMI. The possibility of psychiatric illness in patients with a terminal physical illness is a prominent counter-argument against MAID in general because of fears that such patients may view their situation as unduly negative, thus influencing or even precipitating their wish for MAID (6). For example, it is often argued that as well as underestimating their chances of remission, depressed patients may often feel guilty for being depressed and may consider themselves completely worthless. Beck's influential cognitive theory of depression claims that automatic, spontaneous, and seemingly uncontrollable negative thoughts and negative self-schemas in depressed persons promote an irrationally negative view of themselves, their future and the world in general (40). This can lead to a sense of disgrace and loss of dignity, and to a feeling or conviction that one is a burden to others. The interpersonal psychological theory of suicidal behavior views perceived burdensomeness as a major component of “suicidal desire,” which corresponds more or less to suicidal ideation (21, 24). In their review of the concept, Hill and Pettit (21) claimed there is “[…] support for the incremental validity of perceived burdensomeness as a predictor of suicidal ideation beyond the effects of other well established risk factors” (p. 636). From this perspective, it has been argued that contemporary mental healthcare efforts to prevent suicide should include a focus on perceived burdensomeness (21, 22, 25, 27).

## Should Perceived Burdensomeness Be a Reason for Refusing MAID to Persons with SPMI?

If SPMI renders one's life unlivable, should perceived burdensomeness as a component of that intolerable suffering be considered a reason to deny access to MAID? As mentioned in the Introduction, there are several ways of looking at perceived burdensomeness. If a patient perceives themselves as a burden but is unconcerned about this, any perceived burdensomeness would not contribute to intolerable suffering or contribute to their desire for MAID. On the other hand, a patient might wish to free relatives and the society from this perceived burden or might experience this as an external pressure, rendering their decision less than completely autonomous. Additionally, if a chronic need for care and dependence diminishes the individual's sense of autonomy, perceived burdensomeness may form part of their intolerable suffering.

It follows that perceived burdensomeness *per se* should not be a reason to deny access to MAID. Instead, each patient should be carefully assessed to determine whether perceived burdensomeness is a component of intolerable suffering, and access to MAID should be denied only in cases where a mentally ill person proves incompetent to make this judgment. Of course, this argument is not specific to persons with SPMI, as it can equally apply to those with a somatic illness. However, non-SPMI patients with a somatic illness seem more likely to be able to competently assess the *reality* of the burden on others. Persons with SPMI are more likely to make an exaggerated estimation of their burdensomeness that is a poor reflection of the objective reality—for instance, as a consequence of negative thinking in cases of depression. When these unrealistic perceptions contribute significantly to the individual's understanding of relevant information in deciding to seek MAID, it can be argued that their competence to make that decision is impaired. If that impairment cannot be overcome with appropriate support^2^—for example, by affording an opportunity to reflect on their perceived burdensomeness with a trusted family member—then access to MAID should be blocked.

As incapacity of this kind may be more common among those with SPMI, it is reasonable to ask whether these individuals should be routinely excluded from MAID because of the possibility of perceived burdensomeness. It can equally be argued that it is difficult or impossible for patients or clinicians to say whether perceived burdensomeness is a valid factor independent of their illness. It may also be difficult to determine the extent to which perceived burdensomeness is rooted in the SPMI itself. Persons with SPMI who feel that their suffering has not been effectively alleviated over time may indeed represent a real burden for their relatives. If those family members are involved in decisions about MAID, patients may feel some pressure to choose MAID because of their dependence on housing or financial support (17, 42, 43).

There is also a risk that relatives may unconsciously or unintentionally pressure the patient to seek MAID (17) and that their opinion may play an important role in any such decision, regardless of whether the patient has a mental illness (9, 15, 38). For example, a patient might reject MAID because their family is against this approach (17, 34). In one study of family members of patients who lacked decision-making capacity (DMC), Winter and Parks (44) found that when there was any disagreement, family members tended to favor life-prolonging rather than palliative care approaches and that the decision was influenced more by family members than by the patient's own preference. In some cases, patients might avoid speaking with family members about the fear of being a burden and might overestimate that burden by linking that feeling to the issue of MAID (34). This might lead some patients to refuse MAID because they perceive it as a burden on their families, prompting troubling inner conflict and greater suffering (34). If a patient also perceives themselves as a burden to society as a whole, this may prompt a wish for hastened death (34). For example, some authors have suggested that patients may be inclined to request MAID because they feel they are a financial burden to society, and that legalization of MAID may seem to confirm society's desire to ease that burden (32).

While estimates of their burdensomeness among patients who suffer from severe and persistent depression and other SPMIs may *not* be unrealistic, there is the risk that their judgment may be undermined by negative thinking. Regarding the influence of family or the wider society, it is essential to ensure that the decision to seek MAID is not influenced by external factors but is voluntary (6, 9, 32), and adequate exploration of all these influences and their effects on patient decisions may prove very difficult (32). In this regard, an ethics of care approach offers another perspective on perceived burdensomeness as a relational phenomenon involving the patient, their relatives, their doctor, and society as a whole [e.g., (35, 37)]. These influences on the patient must be considered carefully, as they cannot be completely separated from the patient's subjective perceptions. While no external pressure should be exerted on the patient in making a decision about MAID, the patient must be seen as a relational being who will naturally take account of those relationships in making any such decision. Future research should take fuller account of these issues by integrating this relational component in the discussion about perceived burdensomeness, intolerable suffering, and MAID in patients with SPMI.

Should the discussion about suicidal tendencies be included in the discussion about MAID? As noted earlier, some authors have discussed perceived burdensomeness as a factor in suicide prevention (21, 22, 25, 27). This perspective is particularly important when considering whether patients with SPMI should be allowed to access MAID although possibly perceiving themselves as a burden to others. Psychiatrists are trained to focus on suicide prevention, and this may conflict with acceptance of MAID for patients with mental illness (6, 7, 9, 17, 20, 45–47). For that reason, it seems necessary to question the appropriateness of conflating arguments for suicide prevention with arguments about MAID, as recently discussed in relation to psychiatric disorders (47). Because of certain “*shared characteristics*” (47, 48), one might struggle to distinguish suicide “*in the conventional sense*” (48) from MAID, and emergent expressions like “*rational suicide*” (45, 46) highlight this struggle. We consider it important *not* to conflate these two forms of dying, especially in the context of SPMI.

When perceived burdensomeness is a component of intolerable suffering, the key question is whether a person who is mentally ill can make a competent judgment regarding MAID. If patients with SPMI make unrealistic estimates of their burdensomeness, DMC is impaired. Assuming that this impairment cannot be overcome through supported decision making and that an unrealistic perception of burdensomeness influences the patient's desire for MAID, access to MAID should be denied. For that reason, DMC and perceived burdensomeness should not be assessed as if completely independent of each other. However, this does not mean that impaired DMC regarding MAID inevitably leads to an unrealistic perception of perceived burdensomeness. In general, DMC must be independently re-evaluated for each component of the decision, taking account of both their mutual influence and respective realism. In discussing the question of whether MAID should be allowed on the basis of not wishing to be a burden to others, Bleek (32) concluded that MAID might be permissible for patients with terminal somatic illness. However, he excluded patients with depression because of a possible lack of patient DMC and possible knowledge limitations within the physician community in respect of assessing DMC. It is important to note that presence of a psychiatric disorder *per se* is not sufficient reason to declare a person unable to make a competent decision about MAID (49). It has repeatedly been shown that most psychiatric patients are capable of making decisions about their treatment (50), and that “[…] the prevailing presumption that certain mental disorders are indicative of a lack of decision-making capacity—i.e., automatically render a person incompetent—is persistent but wrong […]” [(2), p. 47].

## Summary and Implications for Practice

We contend that while some individuals with SPMI may experience perceived burdensomeness, this does not mean that all those with SPMI should be routinely excluded from MAID. If SPMI renders a person's life unlivable, perceived burdensomeness as a component of intolerable suffering is not a sufficient reason to deny them access to MAID if they still have DMC. In this latter regard, all requests for MAID and DMC evaluation should be performed to the highest standard by the physicians responsible. Care should also be taken not to introduce medical paternalism “by the back door,” as this would reduce the autonomy of patients with SPMI, who are already particularly vulnerable and stigmatized [see (51)]. Given the nature of the MAID decision, those deemed to have DMC will be capable of a level of self-reflection that includes the ability to take account of their own social networks, relationships, ties, and obligations to the extent that these are relevant. It is also clear that maintaining private or professional relationships often involves dealing with complex and difficult situations, including significant others who might try to persuade one to act in a particular way. Persons with preserved DMC are also capable of reflecting on the feeling or fact of being a burden to others and evaluating this in the context of their own wishes, beliefs, or opinions. The crux of clinical practice in this context is to evaluate whether perceived burdensomeness and a related wish to die are aspects of the underlying SPMI, and whether DMC is compromised to an extent that cannot be overcome by competent supported decision making.

We concede that the risk of incorrectly ascribing incapacity opens the door to routine exclusion of people with SPMI. However, although capacity evaluation in relation to MAID is often a matter of fine judgment, patients with SPMI are not so difficult to evaluate as to warrant a blanket ban. Indeed, we believe that careful assessment in these cases can minimize the risk of error. In any event, avoiding the risk of *any possibility* of error is no more a justification for paternalism when dealing with people with SPMI—including those who report perceived burdensomeness—than for anyone who might request MAID at the end of life.

To ensure careful assessment, Shaffer et al. (17) “[…] strongly encourage jurisdictions that are considering extending (or have extended) PAD [physician assisted dying] to persons with a mental disorder to implement additional safeguards and procedures […].” (p. 149). Advocating a legal framework with stricter guidelines, Vandenberghe (46) made the following recommendation: “[…] even as [a MAID] request is being assessed and processed, recovery-oriented care should continue in parallel. This imperative two-track approach ensures that there is a treatment advocate involved, prevents [MAID] from being used as an escape by an overwhelmed clinician, and keeps the focus of care from being narrowed down to death. Such an approach implies that we don't give up on patients during the long evaluation process. We offer support, do our utmost to reduce suffering and increase comfort, stand by our patients, foster hope, and remain focused on patients' strengths and connectedness with others, including ourselves. We keep striving for a meaningful life and a positive sense of identity, despite the psychiatric illness and its impact” (p. 886f). That being so, we agree with McPherson et al.'s (30) recommendation that the concept of perceived burdensomeness should be incorporated in teaching curricula for professionals working in end-of-life care.

Future research should seek to more precisely quantify the contribution of perceived burdensomeness to intolerable suffering specifically among patients with SPMI, and to what extent this contributes to the desire for MAID. Roest et al. (52), den Hartogh (39), and Bleek (32) stressed the importance of widening the scope of this discussion beyond the patient–therapist relationship and the family circle to the broader sociopolitical context. Rehmann-Sutter (36) noted that “[i]n societies with ‘liberal’ legislation on assisted dying (such as Switzerland, Belgium, and the Netherlands), a duty of the state arises: to safeguard a high level of diligence in the decision-making processes to make sure that everything possible is done to relieve the situation of care by other means than letting the patient take her- or himself out of the equation” (p. 447). According to Shaffer et al. (17), “[t]here are two types of safeguards: direct (i.e., legislation) and indirect (i.e., professional guidelines) […]” (p. 149), and both are of immediate relevance. We believe that, as safeguards and procedures for MAID, professional guidelines should not only include the assessment of intolerable, irremediable suffering and the evaluation of DMC but also, as part of both, perceived burdensomeness, including whether the related wish to die is an integral aspect of the underlying SPMI.

## Conclusion

Perceived burdensomeness is a reality for patients with SPMI and can increase their suffering. Where SPMI renders a patient's life unlivable, perceived burdensomeness as a component of intolerable suffering is not a sufficient reason to deny access to MAID if the patient meets all necessary criteria. We agree with Shaffer et al.'s (17) suggestion for the implementation of additional safeguards and procedures to ensure careful assessment of this vulnerable group when seeking MAID. Aligning with Hodel et al. (47), we recommend that psychiatrists should be involved in the assessment process when a person with SPMI expresses a wish for MAID, especially in cases of perceived burdensomeness, as there is a need to evaluate the extent to which this influences the wish for MAID. Vandenberghe (46) advocated a multidisciplinary approach, especially in cases of “nonterminal illness.” For patients with SPMI, an interdisciplinary consideration of the desire for MAID and the influence of perceived burdensomeness could be beneficial. We further agree with Vandenberghe's (46) suggestion that “[…] recovery-oriented care should continue in parallel […]” (p. 886) with assessment of a request for MAID. In addition, it seems possible that patients with SPMI might benefit from a palliative care approach (4). In cases of perceived burdensomeness, this issue must be addressed in the therapeutic process to alleviate that element of suffering (28, 32, 34, 35, 37), not least because patients have indicated that it is difficult to talk about this with their family (34).

## Author Contributions

JS and MT drafted the manuscript. JS, MT, and CR were involved in conceptualizing the article. All authors were involved in critical revision of the drafted manuscript, and approved the final version submitted for publication.

## Conflict of Interest

The authors declare that the research was conducted in the absence of any commercial or financial relationships that could be construed as a potential conflict of interest.

## Footnotes

^1^In Ruling 03.11.2006 2A.48/, the Federal Supreme Court of Switzerland noted: “Where the wish to die is based on an autonomous and all-embracing decision, it is not prohibited to prescribe sodium pentobarbital to a person suffering from a psychiatric illness and, consequently, to assist him or her in committing suicide…” [6.3.5.1] [translation drawn from *Haas v Switzerland* (2011) ECHR 2422].

^2^According to the UNCRPD, a person cannot be regarded as lacking decision-making capacity unless it proves impossible to gain this capacity with appropriate decision-making support [see, e.g., (41)].
